# Cognitive Impact by Blood Circulating Anti-NMDAR1 Autoantibodies

**DOI:** 10.20900/jpbs.20210009

**Published:** 2021-06-28

**Authors:** Xianjin Zhou

**Affiliations:** Department of Psychiatry, University of California San Diego, La Jolla, 92093, California, USA

**Keywords:** cognitive impairment, anti-NMDAR1 autoantibodies, antigenic epitope

## Abstract

Antibodies persist months and years in blood. Chronic presence of low titers of blood circulating anti-NMDAR1 autoantibodies are sufficient to impair cognitive function in the integrity of the BBB in mice, suggesting potential cognitive damaging effects of low titers of blood circulating anti-NMDAR1 autoantibodies in the general human population and psychiatric patients. Investigation of anti-NMDAR1 autoantibodies against individual NMDAR1 antigenic epitopes may potentially provide risk biomarkers and therapeutic targets for development of immunotherapy as a precision medicine for psychiatric patients in the future.

## INTRODUCTION

*N*-methyl-d-aspartate receptor (NMDAR), a tetrameric multiprotein complex consisting of two obligatory NMDAR1 subunits and two NMDAR2 subunits (2A–2D), is essential for learning and memory as well as many other cognitive functions. Pharmacological studies demonstrated that administration of NMDAR antagonists to healthy persons causes schizophrenia-like symptoms [1]. Recent human genetic studies validated the central role of NMDAR in the development of schizophrenia where truncation of GRIN2A gene (NMDAR2A subunit) carries an odds ratio of 18.1 (3.74–172) for schizophrenia [2]. Aside from genetic mutations in NMDAR genes, physiological and environmental risk factors can also impair NMDAR function. Patients with high titers of anti-NMDAR1 IgG autoantibodies in their brain develop anti-NMDAR1 encephalitis that exhibits many prominent psychiatric symptoms in addition to neurological symptoms [3,4]. As a matter of fact, anti-NMDAR1 encephalitis is often misdiagnosed as schizophrenia or other related psychiatric disorders, further supporting impaired NMDAR neurotransmission in the pathophysiology of psychiatric disorders.

Low titers of anti-NMDAR1 autoantibodies are commonly found in the general human population. Several studies using cell-based assays reported that ~5–10% of the human population, regardless of healthy persons or psychiatric patients, carries low titers of anti-NMDAR1 autoantibodies in their blood [5,6]. Most of these autoantibodies are either IgM or IgA isotype with IgG less frequent [6,7]. These anti-NMDAR1 autoantibodies inhibit NMDAR functions in both in vitro human neurons [8] and in vivo mouse models [5]. In healthy rodents and humans, blood circulating antibodies are mostly blocked from entering brain tissue by the blood-brain barriers (BBB), however, ~0.1–0.2% of the antibodies can cross the BBB into brain tissue regardless of antibody specificities [9-11]. This raises a key question as to whether chronic presence of low titers of blood circulating anti-NMDAR1 autoantibodies may impair human cognitive function and contribute to development of psychiatric symptoms in the integrity of the BBB. In this new emerging field, several significant questions however remain to be fully addressed:

## DETECTION OF LOW TITERS OF BLOOD ANTI-NMDAR1 AUTOANTIBODIES

**Figure 1.**
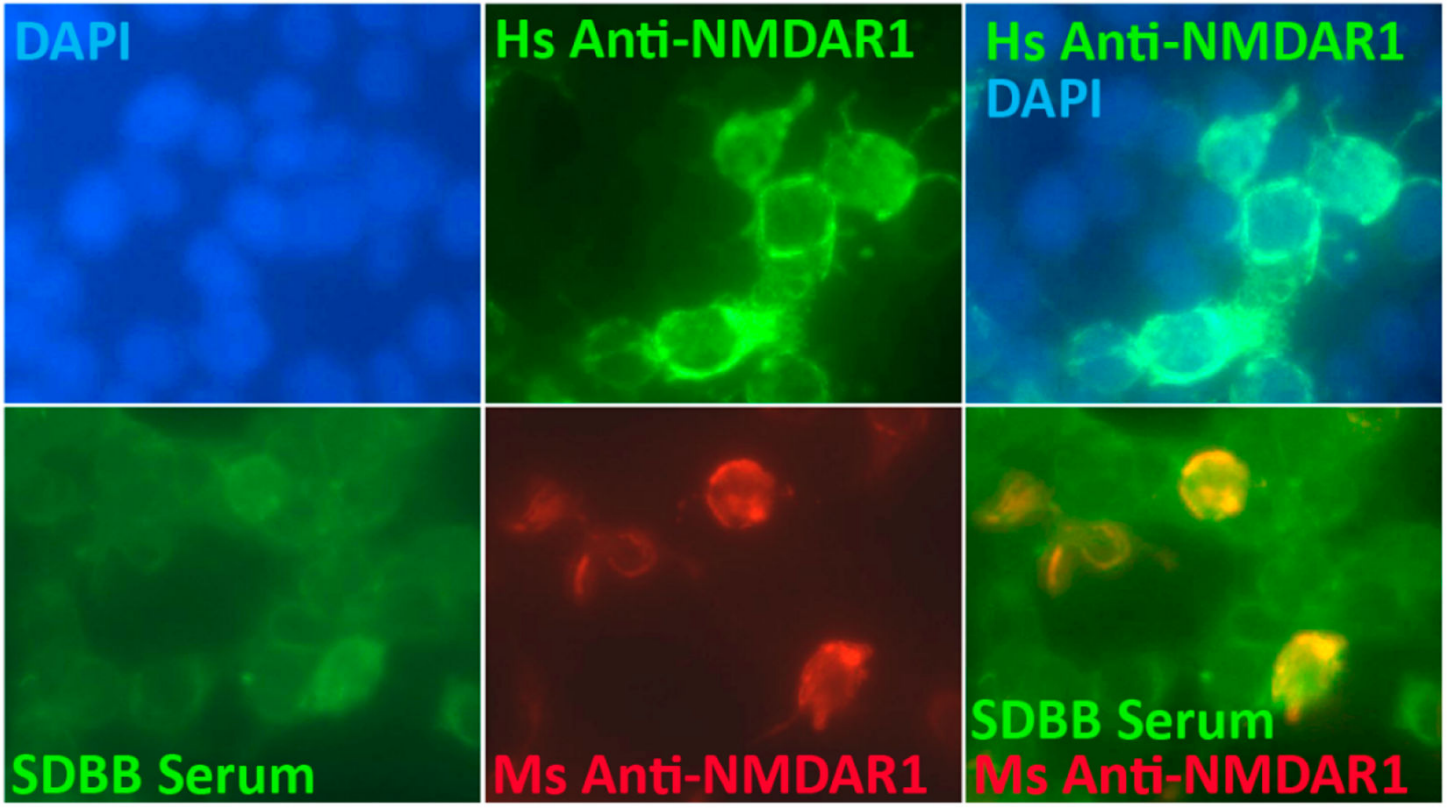
Co-immunocytochemical staining of anti-NMDAR1 autoantibodies using cell-based assays. Human NMDAR1 proteins were expressed on HEK293 cells on BIOCHIPs purchased from Euroimmun. Anti-Human NMDAR1 autoantibody (Euroimmun) recognizes the NMDAR1 proteins on HEK293 cells in cell-based assays (top panel). Co-localization of the staining between human serum (diluted at 1:50) from San Diego Blood Bank (SDBB) and control mouse anti-NMDAR1 autoantibodies (bottom panel), suggesting the presence of low titers of anti-NMDAR1 autoantibodies in the general human population.

In anti-NMDAR1 encephalitis, high titers of anti-NMDAR1 autoantibodies are usually detected using both cell-based assays (Euroimmun) and immunohistochemical staining of rodent brains. Low titers of blood anti-NMDAR1 autoantibodies in the general human population and psychiatric patients, however, are almost solely determined by the cell-based assays in the published studies. We examined low titers of anti-NMDAR1 autoantibodies in human sera collected by San Diego Blood Bank using the cell-based assays (Figure 1). Due to low antibody concentrations, we found that the cell-based assays often suffer from high nonspecific background, and low titers of anti-NMDAR1 autoantibodies can only be discerned by co-immunocytochemical staining with positive control mouse anti-NMDAR1 antibodies. Such co-immunocytochemical staining should be conducted in analysis of low titers of blood anti-NMDAR1 autoantibodies. In addition, a new sensitive assay with less background needs to be developed for detection of low titers of blood anti-NMDAR1 autoantibodies. We recently developed a novel One-Step assay that has little background for detection of blood antibodies [12,13], and confirmed the presence of low titers of blood anti-NMDAR1 autoantibodies in the human sera from San Diego Blood Bank.

## COGNITIVE IMPACT BY LOW TITERS OF BLOOD ANTI-NMDAR1 AUTOANTIBODIES

Chronic effects of low titers of blood anti-NMDAR1 autoantibodies on human cognitive functions have not been well investigated, given that antibodies persist months and years and a small percentages of blood circulating antibodies can infiltrate the BBB into brain parenchyma. We generated mice carrying low titers of blood anti-NMDAR1 autoantibodies (IgM and IgG) against a single NMDAR1 antigenic epitope via active immunization [13]. We found that these mice are healthy and did not display behavioral or cognitive abnormalities except for a profound deficit in spatial working memory and/or novelty detection in the integrity of the BBB across all 3 different mouse cohorts. Although NMDARs are critical for context memory [14,15], we did not find deficient contextual fear memory in mice carrying the anti-NMDAR1 autoantibodies in blood. How the autoantibodies specifically impair spatial working memory but not other NMDAR-modulated behaviors remains to be investigated. It is possible that epitope specificity of the anti-NMDAR1 autoantibodies may contribute to the restricted cognitive impairment. Differential infiltration and retention of anti-NMDAR1 autoantibodies in different brain regions as well as specific impact on nanoscale distribution of synaptic and extrasynaptic NMDARs may also generate different cognitive deficits. Since IgM anti-NMDAR1 autoantibodies are the common antibody isotype in the general human population and psychiatric patients, it is necessary to investigate whether blood IgM anti-NMDAR1 autoantibodies alone are sufficient to cause deficient spatial working memory in the integrity of the BBB. Such studies may provide novel insights to our understanding of effects of low titers of blood circulating anti-NMDAR1 autoantibodies on human cognitive function.

## EPITOPE-SPECIFIC ANTI-NMDAR1 AUTOANTIBODIES

Anti-NMDAR1 autoantibodies have been extensively investigated in patients with anti-NMDAR1 encephalitis and the general human population; however, their antigenic epitopes have not been well characterized. Anti-NMDAR1 autoantibodies were reported to act as NMDAR antagonists [8]. Since anti-NMDAR2A autoantibodies binding a specific antigenic epitope can function as an agonist [16], it is possible that anti-NMDAR1 autoantibodies binding different epitopes may alter NMDAR functions differently and thereby cause different behavioral and cognitive deficits. Consistently, different behavioral abnormalities were observed between our mouse model [13] and two other mouse models carrying anti-NMDAR1 autoantibodies [17,18]. In addition, neuroinflammation was reported in Jones’ mouse model [17] but not ours [13]. Therefore, it is essential to investigate functions of anti-NMDAR1 autoantibodies at the level of individual antigenic epitopes.

In summary, low titers of blood circulating anti-NMDAR1 autoantibodies are sufficient to impair specific cognitive function in the integrity of the BBB in mice, suggesting potential cognitive damaging effects of low titers of blood circulating anti-NMDAR1 autoantibodies in the general human population and psychiatric patients. Investigation of anti-NMDAR1 autoantibodies against individual NMDAR1 antigenic epitopes may potentially provide risk biomarkers and therapeutic targets for development of immunotherapy as a precision medicine for psychiatric patients in the future.
